# The effectiveness of skilled breathing and relaxation techniques during antenatal education on maternal and neonatal outcomes: a systematic review

**DOI:** 10.1186/s12884-022-05178-w

**Published:** 2022-11-19

**Authors:** Vanessa Leutenegger, Susanne Grylka-Baeschlin, Frank Wieber, Deirdre Daly, Jessica Pehlke-Milde

**Affiliations:** 1 School of Health Sciences, Institute of Midwifery, ZHAW Zurich University of Applied Sciences, Winterthur, Switzerland; 2 Faculty of Medicine, University of Zurich, Zurich, Switzerland; 3 ZHAW Zurich University of Applied Sciences, School of Health Sciences, Research Institute of Midwifery, Winterthur, Switzerland; 4 ZHAW Zurich University of Applied Sciences, School of Health Sciences, Research Institute of Public Health, Winterthur, Switzerland; 5 Department of Psychology, University of Konstanz, Constance, Germany; 6 School of Nursing and Midwifery, Trinity College Dublin, Dublin, Ireland

**Keywords:** Antenatal classes, Childbirth preparation, Breathing exercise, Maternal, Neonatal and birth outcomes

## Abstract

**Background:**

Several studies have investigated the relationship between antenatal education classes and pregnancy outcomes. These studies have shown positive effects on mothers, such as a lower epidural rate in the intervention groups. However, until now, the impact on outcomes for mothers and newborns of antenatal education classes that focus on breathing and relaxation techniques has not been examined.

**Aim:**

Investigate the effects of skilled breathing and relaxation techniques provided in antenatal education classes on maternal and neonatal birth outcomes.

**Methods:**

The protocol for this study was registered with PROSPERO (ID: CRD42020192289). A systematic literature search was undertaken and completed in January 2022, using the databases MEDLINE, CINAHL, clinicalTrials.gov, Cochrane Library, Embase and MIDIRS according to a priori formulated PICO criteria: population (pregnant women), intervention (antenatal education classes with integrated breathing and relaxation techniques), comparison (antenatal education classes that do not include skilled breathing and relaxation techniques), and outcome (maternal and neonatal outcomes). The quality of the studies was assessed by two reviewers using the standardised instruments RoB 2 and ROBINS-I.

**Results:**

Ten studies were included in this review, nine randomised controlled trials and one quasi-experimental study. The results indicate that skilled breathing and relaxation techniques may positively influence self-efficacy, the need for pharmacological support, specifically the use of epidural anaesthesia, and the memory of labour pain. No effects were found in relation to predefined neonatal outcomes. The quality of evidence on maternal and neonatal outcomes is inconsistent across studies, as different antenatal education classes with varying interventions, including breathing and relaxation techniques, were offered in the studies.

**Conclusions:**

Women who attended an antenatal education class with breathing and relaxation techniques appear to benefit from the intervention. This applies to the practical implementation and use of breathing and relaxation techniques during labour, increased self-confidence and self-efficacy, and a increased feeling of being in control during labour. This demonstrates the importance of information provision and a focus on breathing and relaxation techniques in antenatal education.

**Supplementary Information:**

The online version contains supplementary material available at 10.1186/s12884-022-05178-w.

## Background

Birth preparation has been offered to pregnant women for several decades and is now mostly offered as part of maternity care during pregnancy [1]. The earliest theoretical approaches identified in the literature were natural childbirth by Dick-Read (1933) and psychoprophylaxis methods by Lamaze (1958). Both approaches emphasise physical and mental health and well-being, physical fitness, knowledge of the physiological processes of labour and birth, and support from a known person/midwife. Currently, there is considerable variation in the organisation and content of antenatal education classes [2]. The choice for women ranges from short classes lasting 2–4 h, to weekend only classes, to antenatal education lasting several weeks. The information offered about birth, pain management, physical activity and especially breathing and relaxation techniques varies considerably [3]. However, two core elements are commonly found: 1. information about pregnancy, birth and the postpartum period, and 2. breathing and relaxation techniques in preparation for labour and birth [2].

Women attend an antenatal education class to be informed and to prepare themselves physically as well as psychologically for labour. An Iranian study shows that women are more likely to believe that they will cope well with labour if they feel well prepared and supported [4]. In addition, women appear to benefit from antenatal education classes that provide them with coping skills, enable them to learn their own strategies and increase their confidence in their abilities [5]. Furthermore, providing information that supports women’s autonomy and active decision-making regarding pain management and stress reduction was found to have positive impact on women's anxiety, fear and hormone release during birth [6]. Finally, according to a qualitative review, breathing and relaxation techniques increase self-confidence, improve the ability to cope with the labour pain, and increase well-being during and after birth [7].

The best available evidence on the effectiveness of antenatal education classes comes from research on women who fear childbirth. In this group of women, group psychoeducation coupled with skilled breathing and relaxation techniques had positive effects on pregnancy outcomes and the women's childbirth experience [8]. Moreover, a randomised controlled trial showed that antenatal education and practised breathing and relaxation were feasible and effective in strengthening the resources of women with increased fear of childbirth and enabling them to act competently and proactively during labour [9]. Studies have also found that not only women who fear childbirth benefit from the relieving effects of breathing and relaxation on labour pain and anxiety, but also expectant first time mothers in general [10, 11]. This applies in particular to skilled breathing and relaxation techniques which are taught in the antenatal education classes and practised at home [12, 13].

There are few studies on the link between birth preparation and neonatal outcomes. In the systematic review by Fink et al. [14], breathing and relaxation techniques during the antenatal period were found to have a positive impact on birth weight and preterm birth rate. The examined interventions involved different active and passive relaxation exercises such as several weeks of active relaxation sessions or mindfulness-based sessions, or massage therapy for relaxation [14]. However, more research is needed.

Birth preparation and thus also the knowledge from the classes is reflected during labour and birth. A greater awareness of breathing and relaxation techniques would be beneficial in order to show women how these skills might help during birth. There is already knowledge about breathing and relaxation techniques in which women have been trained in antenatal education classes [15]. In summary, there is initial evidence that breathing and relaxation techniques as part of antenatal education classes can have a positive impact on maternal and neonatal outcomes, especially in women with increased levels of anxiety. However, little is known about the impact of breathing and relaxation techniques on outcomes for healthy pregnant women with no fear of childbirth or who have no medical or obstetric risks. Therefore, the aim of this systematic review was to investigate the effects of breathing and relaxation techniques taught in antenatal education classes on maternal and neonatal outcomes.

## Methods

### Search strategy

This review was conducted in accordance with the Preferred Reporting Items for Systematic Reviews and Meta-Analyses (PRISMA) guidelines [16]. In July 2020, a systematic review protocol was registered with the international prospective registry for systematic reviews, PROSPERO (registration number CRD42020192289). A systematic literature search was conducted in MEDLINE, CINAHL, ClinicalTrials.gov, the Cochrane Library, Embase and MIDIRS in July 2021 and updated in January 2022. The following keywords were used: “antenatal preparation OR “childbirth education” OR “prenatal education” AND “birth outcomes” OR “pregnancy outcomes” OR “maternal outcomes” OR “neonatal outcomes” (Supplementary material). No date or language limits were applied.

### Study selection

We considered all randomised controlled trials (RCTs), non-randomised and quasi-experimental studies reporting antenatal education classes focusing on breathing and relaxation techniques and no other alternative elements included like aromatherapy or acupressure.

### Population

Healthy pregnant women (primiparous and multiparous) with singleton low-risk pregnancies receiving routine antenatal care and planning a vaginal birth.

### Intervention

The predefined criteria included all group or individual antenatal education classes with integrated breathing and relaxation techniques and exercises that were either taught and practised as interventions in the classes or could be practised at home with instructions.

### Comparison

Antenatal education and preparation without focusing on breathing and relaxation techniques and without exercises.

### Outcome

The following maternal and neonatal outcomes were defined: women's satisfaction with labour and birth experience, duration of labour, pain levels, nedd of pharmacological support for pain management, mobility during labour, mode of birth, fetal blood sampling (fetal capillary blood pH), and Apgar Score at 5 min.

### Study selection

A two-step independent screening process was used to identify studies for inclusion: first citations were screened by title and abstract, then by full-text (VL and SG-B). Disagreements in both phases of the screening were resolved through discussions to achieve consensus. Covidence® was used for all screening, data extraction and quality assessment.

### Data extraction

Two authors independently extracted data from the included trials using a purposively designed form. Data were extracted on study characteristics (e.g., RCT or quasi-experimental study), study setting (e.g., city or geographical region), study participants (e.g., age, parity), detailed description of the intervention (e.g. breathing techniques and exercises, frequency of exercise, partner involvement), mode of delivery of the intervention (e.g. provider, location), time of starting of antenatal intervention (week of pregnancy, trimester) and duration of the intervention (how many weeks, number of days of antenatal class conducted, and number of hours), maternal (self-efficacy, birth experience, duration of labour, pain levels, need of pharmacological support for pain management, mobility during labour, and mode of birth) and neonatal (fetal blood sampling and Apgar Score) outcomes. Differences were resolved in discussions.

## Results

**Fig. 1:**
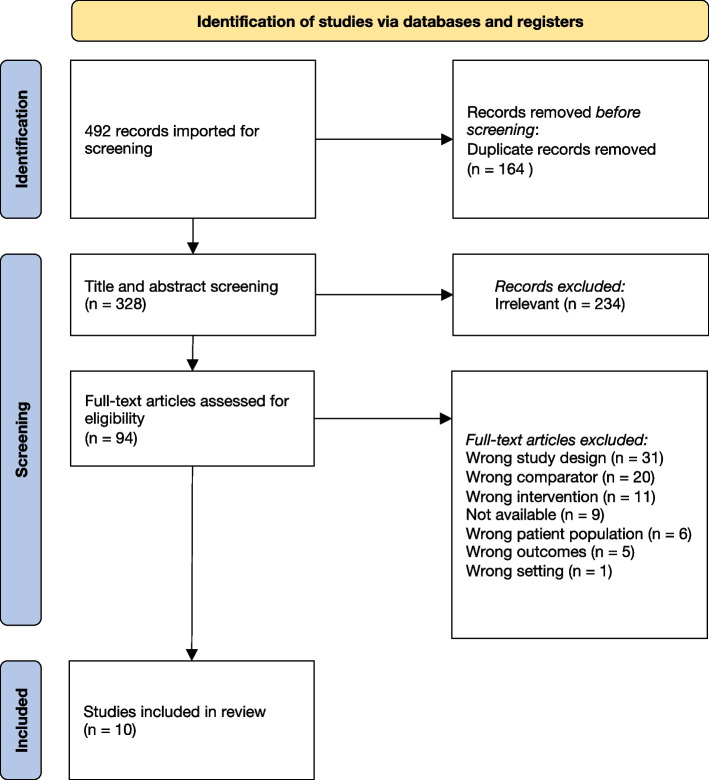
Flow-Chart of literature search (PRISMA)

A total of 328 citations were retrieved following removal of duplicates, and ten studies which were published in eleven articles were eligible for inclusion (Fig. 1). One study published two papers with different research focus, which is why both articles were included [17, 18].

The studies were conducted in eight countries between 1979 and 2016. Of these, nine were RCTs and one was a quasi-experimental study. Two studies were conducted in the USA [19, 20], two in India [21, 22], one in Taiwan [23], one in New Zealand [24], one in Australia [25], one in Brazil [26], one in Iran [17, 18], and one in Sweden [27].

### Description of included trials

A summary of the characteristics of the ten included studies is provided in Table 1. The antenatal education classes in the included studies were highly diverse as were the outcomes examined and measurement instruments used. Furthermore, the diversity of approaches in birth preparation and the information offered, as well as the different concepts and integrated bodywork, such as breathing techniques and exercises, made meta-analysis impossible to conduct.

**Table 1:** Description of included studies

Study	Design	Sample population, N		Description of the intervention	Description of breathing technique	Duration and Frequency	Start time	Outcomes	
		IG	CG					Maternal	Neonatal
**Abbasi, 2021 **[18]	RCT	software = 50 booklet = 51	52	• Education about position modification during pregnancy • Stretching exercises • Breathing techniques and exercises • Relaxation and lower back massage (both software and booklet), weekly telephone contact to remind the participants	Breathing techniques and exercises (no further information)	NI	30–36 weeks	Childbirth self-efficacy (CBSEI), Memory of labour pain (VAS)	
**Bergström, 2009 **[27]	RCT	Women = 544, Men = 529	Women = 543 Men = 534	• Preparation for natural childbirth • Information about non-pharmacological methods for pain relief • Partner’s role as a coach during labour • Psychoprophylactic training between sessions was encouraged and a booklet to facilitate homework was distributed	Practical training in breathing (30 min); homework practicing breathing/relaxation	8 h (4 two-hour sessions during pregnancy, 1 follow-up session within 10 weeks after delivery); 2 h weekly session	3rd trimester	Childbirth experience (W-DEQ A/B), Memory of labour pain (VAS), Pain medication, Mode of birth	
**Duncan, 2017 **[19]	RCT	15	14	• Participants are guided to reframe childbirth pain as unpleasant physical sensations that come and go, moment by moment • Participants are taught how to uncouple the sensory component of pain from its cognitive and affective components, with the objective of decreasing fear and suffering related to the physical pain of childbirth • Participants learn how to be more aware of their own body and fearful reactivity to pain by practicing mindful coping with pain through a pain induction activity with ice • Pregnant women and their birth partners develop personalized strategies to best cope interpersonally and provide support to each other throughout the birth process. Additionally: pain coping strategies, such as mindfulness of breath, partner touch, body movement, and “sounding”	Mindfulness breath, practice at home with audio and handouts	In total 18 h; A short, time-intensive 2.5-day weekend workshop	Late 3rd trimester	Childbirth experience (W-DEQ A/B), Childbirth self-efficacy (CBSEI), Pain medication	
**Howarth, 2019 **[24]	RCT	Overall = 137		The Pink Kit Method for Birthing Better®: Self-taught methodology, anonymised version on the internet, 4 books, 2 audio CD`s, one video	Exercises directed breathing, map pelvis, deep touch relaxation	40 h of content; 50% had to be completed	Recommended start time at 24 weeks’ gestation	Childbirth self-efficacy (CBSEI)	
**Karkada, 2017 **[21]	RCT	270	270	• Antepartum breathing exercise • A video on antenatal breathing exercises was shown (duration approx. 6 min) • Breathing exercises were shown repeatedly in individual classes	Home exercises, daily or twice daily and continued during active phase of first stage of labour	Assessed once at 36 weeks’ gestation	36 weeks’ gestation		Birthweight < 2500 g and > 2500 g
**Levett, 2016 **[25]	RCT	85	87	• Natural state of relaxation (visualisation, breathing, massage, yoga), and facilitate labour progression (yoga, acupressure) and pain relief (breathing, acupressure, visualisation • Education about the physiology of normal birth • Partner support	4 breathing techniques were introduced: soft sleep breaths for relaxation between contractions; blissful belly breaths (BBs) which were used during contractions for pain relief; Cleansing Calming Breaths used following contractions during the transition period of labour; and the gentle birthing breath (GB) which was for use during the second stage of labour and encouraged descent of the baby avoiding active pushing and protection of the pelvic floor	2 days; once	Prior to 36 weeks	Duration of labour, Pain medication, Mode of birth,	5^th^ min Apgar score > 7
**Miquelutti, 2013 **[26]	RCT	Main outcome = 97	Main outcome = 100	Women participated in the physical and educational activities of the BPP conducted in addition to routine activities offered at the prenatal clinic, on the same days of the prenatal visits. During the meetings of approximately 50 min women performed nonaerobic exercises of a protocol adapted for pregnancy and designed to attempt to reduce back pain, possibly to help venous return and to prevent UI and minimize anxiety. Participants received a guide with the exercises to be performed daily at home, consisting of pelvic floor muscle training (PFMT) including rapid (30 times) and sustained maximal contractions (20 times holding for 10 s), stretching exercises to reduce back pain and exercises to improve venous return in the lower limbs	Information about breathing exercises for birth. Relaxation at home: Training of breathing techniques for contraction control during labour, Training of breathing techniques for contraction control during labour; progressive relaxation techniques; massage; mentalisation	monthly from 18–24 weeks until 30 weeks’ gestation; fortnightly till 36 weeks; Duration 50 min	18–24 weeks’ gestation	Duration of labour, Memory of labour pain (VAS), Mode of birth	1^st^ min and 5^th^ min Apgar score > 7, Birthweight > 2500 g
		Secondary outcome = 78	Secondary outcome = 71						
**Pan, 2019 **[23]	RCT	35	39	• Adaptation of MBCP by Nancy Bardack • Transformative experience of pregnancy, childbirth, and postpartum-related adjustments in self-awareness training • Listen to programme-related audio recordings at home six times a week for 30 min each	Three-minute breathing space	8 weeks; Series of nine three-hour classes held once per week and one seven-hour day of silent-meditation practice	After recruitment between 13 and 28 weeks’ gestation	Childbirth self-efficacy (CBSEI)	
**Prince, 2015 **[22]	Quasi-experimental	300	300	• Antenatal exercises by video training (animated and easily understandable) • Antenatal techniques included breathing exercises, relaxation exercises and pelvic floor muscle exercises • Information about pregnancy and labour, benefits of selected antenatal exercise, types of exercises, selected exercises during pregnancy and labour • Each primigravid women was given a record sheet to record the exercises performed at home with relevant instructions and a compact disk (CD) was provided for their practice	NI	34 weeks up to birth; Once (45 min), practice at home (< 200 up to > = 400 h)	NI	Memory of labour pain (VAS)	
**Timm, 1979 **[20]	RCT	31	CG = 40 TAU (no class) = 47	• The delivery process, anatomical, emotional and physical changes in labour • Relaxation and chest-breathing patterns for use in labour • Medications used in labour •Emotional and physical changes in postpartum • Tour of hospital • Combination of lectures, discussions, films and role-playing situations • Use of self-learning programs available in waiting rooms	NI	6 weeks; 10 series	NI	Duration of labour	

*NI* No information, *IG* Intervention group, *CG* Control group

Of the ten included studies, five studies evaluated the maternal outcomes childbirth experience and self-efficacy [17, 19, 23, 24, 27] and four studies examined memory of labour pain [18, 22, 26, 27]. The use of pain medication [19, 25, 27] and the mode of birth were investigated in three studies [25–27]. Duration of labour was also analysed in more detail in three studies [20, 25, 26]. Only one study analysed both defined neonatal outcomes, the Apgar score and birth weight [26]. Levett et al. [25] analysed only the 5 min Apgar score and Karkada et al. [21] only the birth weight.

Inclusion criteria for participants in selected studies were comparable: (1) primiparous or multiparous women, (2) low risk pregnancies with no to low fear of childbirth, (3) second or third trimester and (4) planning a vaginal birth. Six studies included primiparous women only [17–19, 22, 23, 25, 27]. One study examined both primiparous and multiparous women [21] and three studies did not report on parity [20, 24, 26].

### Assessment of study quality

The quality of the studies was assessed using two methods. The standardised quality assessment tool RoB 2.0 was used to assess the quality of the randomised controlled trials [28] and the quality assessment of the non-randomised included articles was conducted using the validated tool ROBINS-I [29]. Both tools consist of several components including representativeness of participants for the target population (selection bias), control of confounding factors, blinding of outcome assessors and participants, reliability and validity of data collection instruments, and number and reasons for withdrawals and dropouts.

**Fig. 2:**
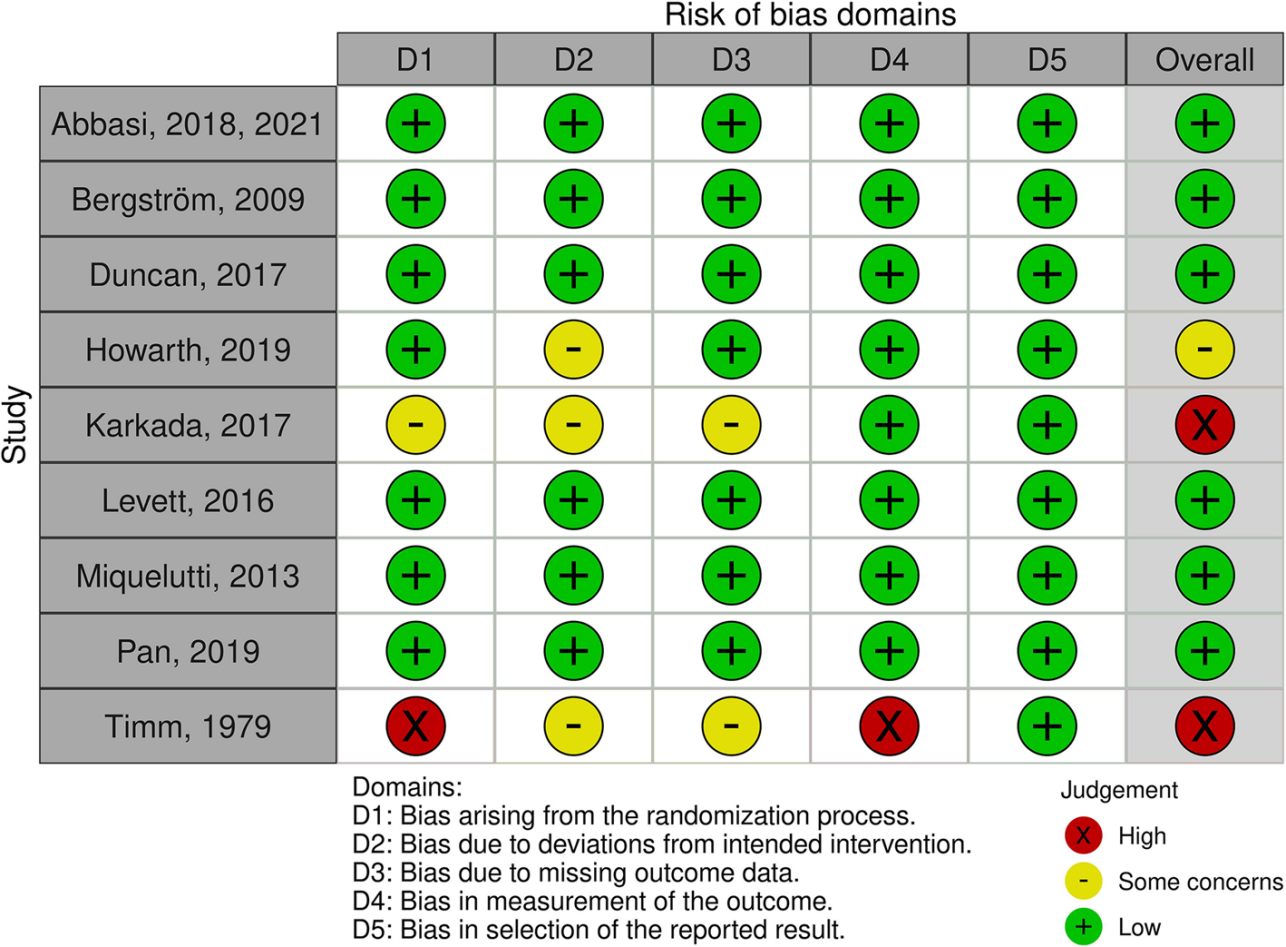
Summary of risk of bias assessment for randomised controlled trials according to RoB 2.0

**Fig. 3:**
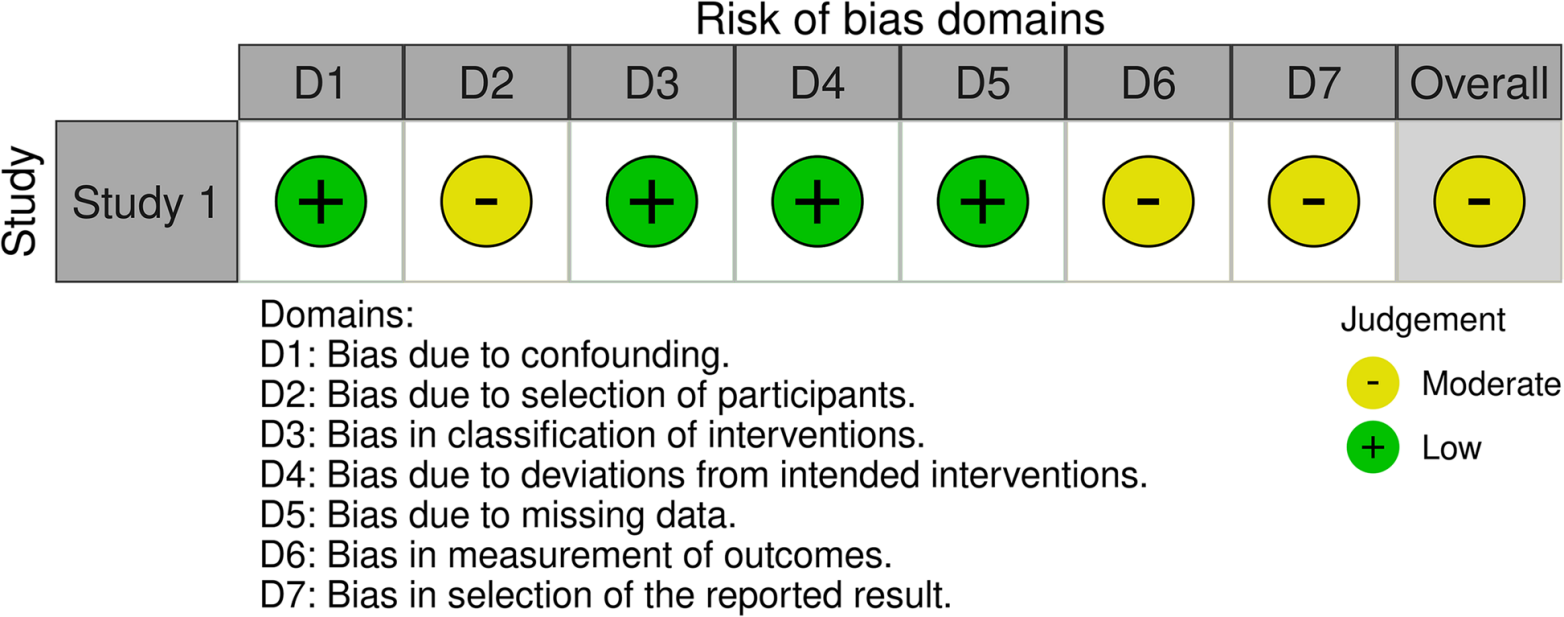
Summary of risk of bias assessment for non-randomised studies according to ROBINS-I

Finally, an overall rating was calculated based on the individual scores. Of the controlled studies, one study was of moderate and two of low quality and did not include specific information on blinding or withdrawal group analysis (Fig. 2). The quality of the non-randomised trial was moderate due to the lack of blinding on outcome measures and withdrawal (Fig. 3).

### Breathing and relaxation techniques

The antenatal education classes were heterogeneous. Common to all courses, however, was the emphasis on repetition of the breathing and relaxation techniques practised. Some classes repeated the exercises during all the antenatal classes, while others asked the women to continue practising the exercises at home (Table 1). The breathing and relaxation techniques used in the ten studies also differed in terms of when the classes were offered (i.e. which trimester), the duration and frequency of use.

Three studies recommended starting the antenatal education classes, including breathing and relaxation techniques, in the third trimester of pregnancy [19, 21, 27], four studies in the second trimester [23–26] and three studies lacked information on the recommended starting time [20, 22] (Table 1). Five studies did not describe the breathing and relaxation exercises taught [17, 18, 20, 21, 24, 27]. Four studies described the breathing and relaxation exercises to some extent. Duncan et al. [19] worked with mindfulness breathing, Howarth et al. [24] with directed breathing, Miquelutti et al. [26] focused on breathing techniques for contraction control and Pan et al. [23] worked with breathing exercises for meditation. Five studies described the different breathing exercises used in detail. Levett et al. [25] included breaths for relaxation between contractions; during contractions for pain relief; during the transition period of labour; and during the second stage of labour. Prince et al.’s intervention included cleansing breathing, slow rhythmic breathing, shallow breathing, passive relaxation, modified breath holding technique and pelvic floor exercises which, the authors stated, helped the mother to relieve the labour pain with significant maternal and fetal well-being [22]. Three studies incorporated breathing techniques from clearly structured and validated programmes such as Mind in Labour (MIL), Mindfulness-Based Childbirth and Parenting Programme or the Pink Kit Method for Birthing Better [19, 23, 24].

### Effects of interventions including breathing techniques on maternal outcomes

#### Childbirth experience

Two studies investigated the childbirth experience [25] (Table 2). Bergström et al. [27] focused on natural childbirth and experience. Participants attended four 2-h weekly sessions and were informed about the childbirth process as well as about pharmacological and non-pharmacological methods of coping with labour pain. However, the reported childbirth experience in the intervention group was similar to the one in the control group (IG: *M* = 49.6, *SD* = 26; CG: *M* = 50.1, *SD* = 25, *p* = 1.0). Also, Duncan et al. [19] reported a not significant difference in the childbirth experience in the two groups (post-birth: IG: *M* = 61.6, *SD* = 20.8; CG: *M* = 57.1, *SD* = 13.4, *p* = 0.48). They used a short, time-intensive 2.5-day weekend workshop with the aim of teaching mindfulness skills for coping with labour pain and fear (Mind in Labor (MIL) by Nancy Bardacke in their study [19]. The workshop included various mindfulness exercises such as reframing labour pain, mindfulness in everyday life and mindful breathing (a more detailed description is missing).

**Table 2:** Maternal and neonatal outcomes

Component	Author, year	Measurement method	Statistical method	Data
Childbirth experience	Bergström, 2009 [27]	Wijma Delivery Expectancy/Experience Questionnaire, version A and B	Mean (SD) by group Mean difference, p-value	Intervention: 49.6 (26) Control: 50.1 (25) -0.5 (-3.2 to 4.1), 1.0
	Duncan, 2017 [19]	24-item version of the Wijma Delivery Expectancy/Experience Questionnaire, T1 3^rd^ Trimester, T2 post intervention, T3 post-birth)	Mean (SD) by group and timepoints T1-T3	Intervention: T1 = 67.1(23.2), T2 = 58.0 (12.2), T3 = 57.1 (13.4) Control: T1 = 65.7 (11.9), T2 = 62.5 (13.0), T3 = 61.6 (23.2)
Childbirth self-efficacy	Abbasi, 2021, 2018 [17, 18]	Childbirth Self-Efficacy Inventory (CBSEI) T1 pre intervention, T2 post intervention	Mean (SD) by group and timepoints T1, T2, p-value Adjusted mean difference with 95% CI, p-value	Intervention Software: T1 = 141.8 (7.2), T2 = 308.4 (11.3) Intervention Booklet: T1 = 143.3 (7.7), T2 = 262.5 (39.5) Control: T1 = 142.1 (7.5), T2 = 149.1 (23.0) T1 = 0.563, T2 = < 0.001 Software with booklet: T1 = 1.5 (-5.0 to 2.0), 0.574, T2 = 45.9 (33.0 to 58.7), < 0.001 Software with control: T1 = -0.3 (-3.8 to 3.2), 0.981, T2 = 159.3 (146.5 to 172.0), < 0.001 Booklet with control: T1 = 1.2 (-2.3 to 4.7), 0.687, T2 = 113.4 (100.7 to 126.1), < 0.001
	Duncan, 2017 [19]	Childbirth Self-Efficacy Inventory (CBSEI), T1: 3^rd^ Trimester, T2: Post-intervention	Mean (SD) by group and timepoints T1, T2	Intervention: T1 = 165.1 (87.2), T2 = 243.3 (41.6) Control: T1 = 197.3 (49.0), T2 = 212.0 (35.4)
	Howarth, 2019 [24]	Childbirth Self-Efficacy Inventory (CBSEI), T1: 24 weeks gestation, T2: 36 weeks gestation	Mean (SD) by group T1, T2 Mean difference, p-value	Intervention: T1 = 188.63, T2 = 215.21 Control: T1 = 194.85, T2 = 190.81 TAU: T1 = 177.59, T2 = 180.61 Intervention with Control: 24.40, 0.021 Intervention with TAU: 34.60, < 0.001 Control with TAU: 10.21, 0.443
	Pan, 2019 [23]	Chinese Childbirth Self-Efficacy Inventory (CBSEI-C32), T0 pre intervention, T1 post intervention, T2 follow-up 36 weeks gestation	Mean (SD) by group *p*-value B, SE, 95% CI, Wald X, p-value	Intervention: 229.33 (41.76) Control: 213.91 (44.67) 0.08 Intervention vs. Control: 8.18, 4.52, (-0.67 to 17.03), 3.28, 0.07 T1 vs. T0: 6.88, 5.90, (-4.68 to 18.45), 1.36, 0.24 T2 vs. T0: 1.69, 8.14, (-17.64 to 14.26), 0.04, 0.84 Intervention with T1: 26.38, 10.55 (6.24 to 47.61), 6.51, 0.01 Intervention with T2: 26.92, 9.10 (8.54 to 44.23), 8.40, < 0.001
Lenght of labour	Levett, 2016 [25]	First stage (h,min) Second stage (h,min) Total length of labour (h, min)	Mean (SD) by group Mean difference with 95% CI Mean (SD) by group Mean difference with 95% CI Mean (SD) by group Mean difference with 95% CI	Intervention: 6.12 (3.95) Control: 6.53 (3.90) − 0.41 (− 1.79 to 0.98) p = 0.56 Intervention: 1.00 (0.87) Control: 1.32 (0.98) − 0.32 (− 0.64 to 0.002) p = 0.05 Intervention: 7.43 (4.13) Control: 8.20 (4.37) − 0.77 (− 2.26 to 0.72) p = 0.31
	Miquelutti, 2013 [26]	Duration of active phase (min) Duration of delivery (min)	Mean (SD) by group Mean difference with 95% CI Mean (SD) by group Mean difference with 95% CI	Intervention: 284.5 (± 175) Control: 254.2 (± 139.4) 30.3 (− 40.9–101.4) Intervention: 29.2 (± 23.3) Control: 19.7 (± 13) 9.48 (0.32–18.64)
	Timm, 1979 [20]	Total length of labour (h, min)	Mean by group	Intervention: 10.88 Control: 10.06 TAU (no class): 9.19
Memory of labour pain	Abbasi, 2021, 2018 [17, 18]	Visual Analogue Scale (VAS) at 4 stages of cervical dilatation (4,6,8,10)	Mean (SD) by group, SD, p-value	4 cm: Intervention Software: 2.5 (0.8); Intervention Booklet: 2.6 (0.8); Control: 2.6 (0.8), *p* = 0.956 6 cm: Intervention Software: 5.2 (0.7), Intervention Booklet: 5.2 (0.5), Control: 5.1 (0.6), *p* = 0.769 8 cm: Intervention Software: 7.0 (0.8), Intervention Booklet: 7.1 (0.8), Control: 7.1 (0.8), *p* = 0.811 10 cm: Intervention Software: 8.7 (0.8), Intervention Booklet: 8.8 (0.7), Control: 8.7 (0.7), *p* = 0.512
	Duncan, 2017 [19]	Visual Analogue Scale (VAS) at 3-4 cm, 4 cm to pushing, during pushing till birth, from birth to delivery of placenta	Average mean score	Intervention: 5.2 Control: 3.88
	Bergström, 2009 [27]	8-point likert scale (no pain to worst pain)	Mean (SD) by group	Intervention: 4.9 (1.8) Control: 4.9 (1.8)
	Miquelutti, 2013 [26]	Lumbar pain measured with Visual Analogue Scale (VAS) at Baseline T0, intermediate T1, final T2 Pelvic pain measured with Visual Analogue Scale (VAS) at Baseline T0, intermediate T1, final T2	Mean (SD) by group Mean difference with 95% CI, n Mean (SD) by group Mean difference with 95% CI, n Mean (SD) by group Mean difference with 95% CI, n Mean (SD) by group Mean difference with 95% CI, n Mean (SD) by group Mean difference with 95% CI, n Mean (SD) by group Mean difference with 95% CI, n	T0: Intervention: 4.7 ± 2.7 T0: Control: 4.5 ± 2.2 0.23 (− 0.64–1.09), 122 T1: Intervention: 5.1 ± 2.3 T1: Control: 5.1 ± 2.5 0.08 (− 0.86–1.03), 99 T2: Intervention: 5.1 ± 2.3 T2: Control: 4.8 ± 2.5 0.34 (− 0.61–1.28), 102 T0: Intervention: 3.8 ± 2.1 T0: Control: 4.7 ± 2.4 − 0.9 (− 2.49–0.78), 29 T1: Intervention: 4.9 ± 2.7 T1: Control: 5.4 ± 2.3 − 0.47 (− 2.12–1.19), 39 T2: Intervention: 5.5 ± 2.9 T2: Control: 5.9 ± 2.8 − 0.38 (− 2.09–1.33), 44
	Prince, 2015 [22]	Visual Analogue Scale (VAS)	Mean (SD) by group Chi-Square, t-test, *p*-value	Intervention: 7.0 (1.0) Control: 8.8 (1.3) Χ^2^ = 335.0, t = 19.65, p = 0.000
Use of pain medication	Bergström, 2009 [27]	Epidural rates	N (%)	Intervention: 247 (52) Control: 252 (52)
	Duncan, 2017 [19]	Epidural/spinal anesthesia Opioid analgesia	N (%) N (%)	Intervention: 12 (85.7%) Control: 11 (84.6%) Intervention: 4 (30.8%) Control: 8 (61.5%)
	Levett, 2016 [25]	Epidural rates	N (%) RR with 95% CI, p-value	Intervention: 21 (23.9%) Control: 57 (68.7%) 0.35 (0.23 to 0.52), *p* = ≤0.0001
Mode of birth	Bergström, 2009 [27]	Spontaneous vaginal Instrumental Elective caesarean Emergency caesarean	RR with 95% CI, p-value	1.0 (0.9 to 1.1), *p* = 1.0 1.1 (0.8 to 1.6), *p* = 0.4 0.9 (0.6 to 1.6), *p* = 0.8 0.9 (0.7 to 1.2), *p* = 0.5
	Levett, 2016 [25]	Normal vaginal birth C-Section Instrumental	N (%) RR with 95% CI, p-value	Intervention: 60 (68.2%) Control: 39 (47.0%) 1.56 (1.12 to 2.17), *p* = ≤ 0.01 Intervention: 16 (182%) Control:27 (32.5%) 0.52 (0.31 to 0.87), *p* = 0.017 Intervention:12 (13.6%) Control: 17 (20.5%)
	Miquelutti, 2013 [26]	Vaginal delivery	N (%) RR (95% CI)	0.57 (0.30 to 1.09) Intervention: 44 (57.9%) Control: 38 (53.5%) 1.08 (0.81–1.44)
Apgar score	Levett 2016 [25]	5^th^ min Apgar score	N (%) RR with 95% CI, p-value	Intervention: 3 (3.4) Control: 4 (4.8) 0.99 (0.95 to 1.03), *p* = 1.03
	Miquelutti, 2013 [26]	1^st^ min Apgar score ≥ 7 5^th^ min Apgar score ≥ 7	N (%) RR with 95% CI, p-value N (%) RR with 95% CI, p-value	Intervention: 70 (93.3%) Control:63 (92.7%) 1.01 (0.92–1.10) Intervention: 75 (100%) Control: 67 (98.5%) 1.01 (0.99–1.04)
Birth weight	Karkada, 2017 [21]	Birth weight < 2500 g Birth weight ≥ 2500 g	N (%) N (%) OR with 95% CI, p-value	Intervention: 11 (5%), Control: 92 (8%) Intervention: 256 (95%) Control: 229 (92%) 1.389 (0.682–2.833), *p* = 0.365
	Miquelutti, 2013 [26]	Birth weight ≥ 2500 g	N (%) RR with 95% CI, p-value	Intervention: 70 (92.1%) Control: 64 (94.1%) 0.98 (0.90–1.07)

#### Self-efficacy

Four studies examined self-efficacy in relation to antenatal education using the Childbirth Self-Efficacy Inventory (CBSEI) [17, 19, 23, 24]. They all showed that self-efficacy could be increased during pregnancy with interventions including breathing and relaxation techniques (Table 2). Abbasi et al. [17] defined two intervention groups and a control group and showed that self-efficacy was significantly higher in both the booklet group and the software group compared to the control group (post: software group: *M* = 159.3, *95% CI* 146.5 to 172.0; booklet group: *M* = 113.4, *95% CI* 100.7 to 126.1, *p* =  < 0.05). The mean score in the trial by Pan et al. [23] for the experimental group was significantly higher than the comparison group directly after the intervention. Thus, the intervention was successful in increasing self-efficacy.

According to Duncan's study, there was a significant increase in self-efficacy scores post intervention (pre: IG: *M* = 165.1, *SD* = 87.2; CG: *M* = 197.3, *SD* = 49.0; post: IG: *M* = 243.3, *SD* = 41.6, CG: *M* = 212.0, *SD* = 35.4). Similarly, Howarth et al. [20] found that self-efficacy was significantly increased in the intervention group compared to the control group (Pre: mean = 188.63 vs. 194.85; Post: mean = 215.21 vs. 190.81).

#### Mode of birth

The mode of birth was analysed in three randomised controlled trials [25–27]. In Bergström et al. [27] the birth mode was comparable between the intervention and control group (vaginal birth: 66% in both groups, instrumental vaginal birth IG = 14%; CG = 12%, caesarean section IG = 20%, CG = 21.5%). According to the authors, 85% (*n* = 411) of women in the intervention group practised breathing exercises during their pregnancy at home, and 70% of the women (*n* = 331) used the breathing techniques during labour. According to Miquelutti et al. [26], 44 women (57.9%) vs. 38 (53.5%) gave birth spontaneously while in Levett et al. [25], 69 women (68.2%) vs. 39 women (47.0%) experienced a vaginal birth. Thus, the proportion of spontaneous or vaginal births was higher in the intervention groups in Miquelutti et al.’s [26] and Levett et al.’s studies [25].

#### Duration of labour

There was no clear definition of labour duration in any of the included studies, which investigated this outcome. Bergström et al. [27] found a comparable mean labour duration of eleven hours in both groups (SD = 9.9). The RCTs by Miquelutti et al. [26] and Levett et al. [25] analysed the first stage of labour and the second stage of labour separately; the two stages were slightly shorter in the intervention group in Levett et al. [25] (first stage (min) 367.2 vs. 391.8, second stage (min) 60 vs. 79.2, total length of labour (min) 445.8 vs. 492) compared to Miquelutti et al. [26] where the stages took more time in the intervention group than in the control group (length of first stage (min) 284.5 vs. 254.2, length of second stage (min) 29.2 vs. 19.7). Both studies included different pain management techniques, but both spent time on bodywork and taught women various breathing techniques, and relaxation exercises including visualisation and massage methods.

#### Need of pharmacological support

Only Duncan's study investigated the use of opioids. In the intervention group, four out of 13 women chose to use opioids during labour, compared to eight out of 13 women in the control group [28]. Their intervention focused on pain reframing, personal body control, disconnecting the sensory component of pain from the cognitive and affective components, and developing coping strategies with the support person. The use of epidural anaesthesia was examined in three studies [19, 25, 27]. The intervention group in Levett et al. [25] showed significantly decreased epidural use compared to the control group (IG: 21 (23.9%); CG: 57 (68.7%), *RR* = 0.35, *95% CI* = 0.23–0.52, *p* =  < 0.01). In Bergström et al.’s [27] and Duncan et al.’s [28] studies the differences between the two groups were less pronounced (Table 2).

#### Pain levels

The pain level was investigated in four studies [18, 22, 26, 27]. Prince et al. [22] found significantly lower pain level in the intervention group compared to the control group (IG: *M* = 7.0, *SD*, 1.0; CG: *M* = 8.8, *SD* = 1.3, *p* =  < 0.01). However, no statistically significant differences were found in Bergström et al. [27] and Miquelutti et al. [26] (Bergström et al.: IG: *M* = 4.9, *SD* = 1.8 vs. CG: *M* = 4.9, *SD* = 1.8, *p* = 0.7; Miquelutti et al.: lumbar: IG: *M* = 5.1, *SD* = 2.3 vs. CG: *M* = 4.8, *SD* = 2.5; pelvic: IG: *M* = 5.5, *SD* = 2.9, CG: *M* = 5.9, *SD* = 2.8). Only Miquelutti et al. [26] distinguished between lumbar and pelvic floor pain based on their specific intervention performed.

#### Effects of interventions including breathing techniques on neonatal outcomes

Neonatal outcomes were investigated in three studies [21, 25, 26] (Table 2). The reported neonatal outcomes included birth weight and Apgar score. Unfortunately, none of the included studies investigated the outcome fetal blood sampling, which is why no results could be shown here. Neither Miquelutti's trial [26] nor Levett's study [25] reported significant differences in the 5-min Apgar score below seven in outcomes of healthy women between the intervention and control group. Likewise, there was no significant difference in neonatal birth weight across all three studies.

## Discussion

To our knowledge, this is the first systematic review to examine the impact on outcomes for mothers and newborns of antenatal education classes that focus on breathing and relaxation techniques. The results provide evidence that breathing and relaxation techniques improve self-efficacy [17, 19, 23, 24], lower the requirement of pharmacological support – specifically the use of epidural anaesthesia [19, 25, 27] – and reduce the reported pain levels remembered from the labour pain [25–27]. It is important to consider that the quality of evidence on maternal and neonatal outcomes is inconsistent across studies, as different antenatal education classes with varying interventions – including breathing and relaxation techniques and exercises – were the classes offered in the studies.

Results from the studies are of limited use in future development of antenatal education classes. With regard to our defined neonatal outcomes, none of the studies found significant differences between the intervention and control groups. Furthermore, there were no significant differences found between the various antenatal groups included in the studies, with regard to the impact on a range of outcomes, including skilled breathing and relaxation techniques and women`s satisfaction and childbirth experience, duration of birth, mobility during labour and mode of birth.

No evidence was found that skilled breathing techniques and relaxation taught in antenatal classes had an impact on the childbirth experience. This finding is consistent with the systematic review by Hong et al. [1].

This result is expected, given the complexity of ‘birth experience’ and the difficulty, therefore, of accurately measuring outcomes. So far, there is no clear definition of the term "childbirth experience", which is based on different concepts, e.g., women's self-assessment of long-term memories of their childbirth, sense of control, fulfilment of expectations, self-confidence, and involvement in decision-making [5]. Thus, the challenge in having agreement on the definition might be a possible explanation as to why no direct impact on the childbirth experience has been shown so far.

Although there has as yet been no assessable outcome with regard to the impact of breathing and relaxation techniques on the childbirth experience, this does not apply to self-efficacy, which seems to be related to the childbirth experience as such [17, 19, 23, 24]. As a theoretical framework for exploring, explaining, and predicting health behaviours, self-efficacy has been used in a wide range of health promotion research. In birth preparation, self-efficacy is particularly relevant from variety perspectives. Women with higher self-efficacy levels in pregnancy, for example, report having less pain during labour, less fear of childbirth, feel better prepared overall to deal with labour pain and report feeling more in control over painful situations. Self-efficacy improved over time in the intervention groups in all four studies [17, 19, 23, 24]. This may be explained by Bandura's self-efficacy theory (Bandura 1997). Here, two conceptually independent components are important, namely outcome expectancy and efficacy expectancy (Bandura 1986). Outcome expectancy refers to the belief in the likely consequences that a behaviour will result in, while efficacy expectancy refers to a person's perceived ability to perform a behaviour. According to Bandura (1986, 1997), there are four approaches to improving self-efficacy perceptions. These are performance delivery, vicarious experience, verbal persuasion and physiological condition. According to Bandura (1997), a strong belief in one's own efficacy to exercise some control over one's physical state can serve as a psychological predictor of the likely level of health outcomes. This could be a possible explanation for the increased self-efficacy in our results. Women who can actively participate in their labour process and feel actively involved may have a greater sense of being in control than those who are more passively involved [30, 31]. Another possible connection can be observed between self-efficacy and pain intensity. With a strengthened sense of control and self-confidence, which can be achieved through increased self-efficacy, the perception of pain is apparently affected [32].

Our findings indicate that skilled breathing and relaxation techniques during antenatal education classes have an impact on the use of these techniques as well as other skilled non-pharmacological methods such as visualisation to cope with labour pain. Similarly, skilled breathing and relaxation techniques during pregnancy have a positive effect on coping with labour pain [33]. All three studies that investigated the use of epidurals found significantly lower usage in the intervention groups compared to the control groups. [19, 25, 27]. Thomson et al. investigated women's needs during childbirth, what they wanted and how this influenced their preference for pharmacological and non-pharmacological pain relief options. Women who engaged in massage and/or relaxation methods prior to their labour process reported that knowledge of these methods for pain relief provided a sense of relief. Practising non-pharmacological techniques enabled women to feel 'prepared', 'calm' and 'empowered' for birth [7]. The importance of providing information and the opportunity to learn and practice breathing and relaxation techniques are therefore highly relevant in antenatal education.

Furthermore, there seems to be a link between breathing and relaxation techniques and pain intensity or pain levels. Some women who used breathing and relaxation and/or massage techniques reported that these methods helped to make the pain more tolerable [7]. Hassanzadeh et al. also showed similar results in their study. Women who attended antenatal education classes stated that knowledge about pain management and the possibilities of non-pharmacological interventions was very helpful during birth. They indicated that the exercises they learned and the breathing and relaxation techniques they could use during labour were very helpful and enabled them to cope with labour pain [5].

## Strengths and limitations of this study

This systematic review was conducted according to the guidelines of the Cochrane Handbook [34]. Without restriction, the authors attempted to include all possible studies according to the definition. The design of antenatal care and antenatal education varies from country to country and is also influenced by access to health care, the level of which varies depending on location. The WHO and NICE provide recommendations for antenatal education classes, including possible content and the formulation of goals, and these can certainly be considered as a baseline for the development of an antenatal education class. Thus, a certain comparability of the general understanding and requirements of an antenatal education class should be given.

In this review, the interventions and adherence measures were heterogeneous and could not be meta-analysed [35]. Instead, we present (1) simple summary data for each intervention including breathing and relaxation techniques and (2) a summary on each outcome with either risk ratio or mean differences following PRISMA guidelines [36]. The lack of blinding in the included studies is also a limitation. To reduce the response bias, some studies used blinded assessors and all the studies used the self-administered method. To assist in the identification of comparable outcomes, the target population of the selected studies was low-risk pregnant women with no to low fear of childbirth; as these samples do not represent the total population of pregnant women, this may be considered a limitation of this review. The diversity of breathing techniques and exercises as well as the structure, content and frequency of the observed antenatal education classes do not allow any concrete conclusions, nor is it possible to give specific recommendations on breathing techniques and their implementation and application. Likewise, the breathing and relaxation techniques could not be observed alone, as they were taught in combination with other elements in antenatal education. Therefore we recommend that further research be undertaken that includes a clear description of the antenatal classes, as well as the breathing and relaxation techniques practised and the recommendations for independent practice at home.

## Conclusion

Our findings suggest that some of the predefined outcomes were either too complex or unclear, making it difficult to use them for comparison with our actual results. Therefore, no correlation was found between antenatal education classes that include skilled breathing and relaxation techniques and women’s satisfaction, duration of labour, mobility during labour, mode of birth and neonatal outcomes. Given the heterogeneity and quality of the studies included, it is recommended that an antenatal class be developed that is transparent and reproducible. A possible approach for the development of such an intervention could be concepts for the development of a complex intervention [37, 38].

In women who attended an antenatal education class with integrated breathing and relaxation techniques, improved maternal and neonatal outcomes were observed. Antenatal education classes including skilled breathing and relaxation techniques have a positive effect on self-efficacy, the request for pharmacological support – specifically the use of epidural anaesthesia – and the memory of labour pain. This highlights how important it is to provide information and practice breathing and relaxation techniques in antenatal education and and for further research on this topic to be undertaken. 

## Supplementary Information

**Additional file 1.** 

**Additional file 2.**

## Data Availability

All data generated or analysed during this study are included in this published article [and its supplementary information files]. The datasets used and analysed in the presented study are available online. No unpublished data was included.
