# Supplementary material for: The effectiveness of skilled breathing and relaxation techniques during antenatal education on maternal and neonatal outcomes: a systematic review
Source: BMC Pregnancy Childbirth. 2022 Nov 19;22:856. doi: 10.1186/s12884-022-05178-w (PMC9675115; doi:10.1186/s12884-022-05178-w)
Supplement: Supplementary file 1 — Additional file 1. [file 12884_2022_5178_MOESM1_ESM.pdf]

Author, year	Country	Design	Setting	Aim	Sample population, N	Age (mean, y)
Abbasi, 2018, 2021	Iran	Randomized controlled trial	health centres and Fatemeh Al-Zahra Hospital of the Miandoab city-West Azerbaijan Province	To compare the effect of e-learning and the booklet training on the labour pain and anxiety	Intervention group (booklet) N= 50 Intervention group (software) N=51 Controll group N=52	IG (software): 25.5 (3.8) IG (booklet): 25.9 (3.6) CG: 25.1 (3.2)
Bergström, 2009	Sweden	Randomized controlled trial	Fifteen antenatal clinics in Sweden	To determine the effect of antenatal group education on the three outcomes: labour pain expressed as a need for epidural analgesia, overall experience of childbirth and experience of parental stress in early parenthood	Women: Intervention group N=544, control group N=543 Men: Intervention group N=529, control group N=534	Intervention group 28.8, control group 28.6
Duncan, 2017	USA	Randomized controlled trial	Setting not clearly named, San Francisco	To test the hypotheses that mindfulness training through Mind in Labour (MIL) would: 1) produce an adaptive shift in fear and pain-related appraisals of childbirth, thereby increasing childbirth self-efficacy and reducing pain catastrophizing; 2) lead to lower labor pain ratings, less use of pain medication in labor, and greater birth satisfaction; and 3) lower perinatal depression symptoms and protect against postpartum depression	Intervention group N=15, control group N=14	NI
Howarth, 2019	New Zealand	Randomized controlled trial	Nation wide in New Zealand	To discover whether the programme The Pink Kit Method for Birthing Better® would be an effective tool for increasing childbirth self-efficacy in first time mothers	Overall N=137	29.2
Karkada, 2017	India	Randomized controlled trial	outpatient department of obstetrics and gynecology unit of a secondary health care institution of Udupi District	To determine the effectiveness of antepartum breathing exercises and factors responsible for spontaneous vaginal delivery among primigravid and multigravid women.	Intervention group N=270, control group N=270	Intervention group 26.7, control group 26.5
Levett, 2016	Australia	Randomized controlled trial	2 public hospitals in Sydney	To test the hypothesis that nulliparous women who undergo a Complementary Medicine (CM) techniques antenatal education course, in addition to usual antenatal care, would use less epidural block than nulliparous women who receive usual antenatal care alone	Intervention group N=85, control group N=87	Intervention group 28.9, control group 30.4
Miquelutti, 2013	Brazil	Randomized controlled trial	Women ' s Integral Health Care Hospital (CAISM), University of Campinas (UNICAMP) and four municipal primary healthcare centers in Campinas, São Paulo	To evaluate effectiveness and safety of a birth preparation programme to minimize lumbopelvic pain, urinary incontinence, anxiety, and increase physical activity during pregnancy as well as to compare its effects on perinatal outcomes comparing two groups of nulliparous women	Main outcome: Intervention group N=97, control group N=100 Secondary outcome: Intervention group N=78, control group N=71	Intervention group 22.9, control group 22.9
Pan, 2019	Taiwan	Randomized controlled trial	Regional hospital in northern Taiwan (2000 births yearly)	To explore the efficacy of the MBCP programme on prenatal stress, depression, mindfulness and childbirth self-efficacy	Intervention group N=51, control group N=45	Intervention group 32.6, control group 33.0, overall 32.8
Prince, 2015	India	quasi-experimental	One hospital in Karnataka (1300 deliveries yearly)	To determine the effect of selected approved antenatal exercises during labor among primigravid women.	Intervention group N=300, control group N=300	NI
Timm, 1979	USA	Randomized controlled trial	Urban Hospital medical facility, Pennsylvania	To evaluate the effectivness of prenatal education classes on the medication used during labour and on the birth weight	Intervention group N=31, control group N=40, No class gorup (TAU) N=47	NI

Author, year	Description of the intervention	description of breathing technique	Duration of class	Start time of the course	Frequency
Abbasi 2018, 2021	Educations about position modification during pregnancy, stretching exercises, breathing techniques and exercises, relaxation and lower back massage (both software and booklet), weekly telephone contact to remind the participants	NI	from 30-36 weeks till childbirth	30-36 weeks	NI
Bergström, 2009	preparation for natural childbirth, Information about non-pharmacological methods for pain relief, partner's role as a coach during labour. In each session, 30 minutes were spent on practical training in breathing relaxation and massage techniques. Psychoprophylactic training between sessions was encouraged and a booklet to facilitate homework was distributed.	30 minutes were spent on practical training in breathing; homework practicing breathing/relaxation	8 hours (four 2 hour sessions during pregnancy, 1 follow-up session within 10 weeks after delivery)	3. trimester	2hours weekly session
Duncan, 2017	1) participants are guided to reframe childbirth pain as unpleasant physical sensations that come and go, moment by moment; 2) participants are taught how to uncouple the sensory component of pain from its cognitive and affective components, with the objective of decreasing fear and suffering related to the physical pain of childbirth; 3) participants learn how to be more aware of their own body and fearful reactivity to pain by practicing mind-ful coping with pain through a pain induction activity with ice; 4) pregnant women and their birth partners develop personalized strategies to best cope interpersonally and provide support to each other throughou the birth process. Additionally: pain coping strategies, such as mindfulness of breath, partner touch, body movement, and "sounding".	mindfulness breath, practice at home with audio and handouts	18 hours, 2.5 days, weekend workshop	Late 3rd trimester	A short, time-intensive 2.5-day weekend workshop
Howarth, 2019	self-teached methodology, anonymized version on the internet, 4 books, 2 audio CD`s, one video (The Pink Kit Method for Birthing Better),	exercises directed breathing, map pelvis, deep touch relaxation	40hours of contenct	Recommended 24 weeks	50% had to be completed
Karkada, 2017	experimental group received antepartum breathing exercise; experimental group were showed a video on antenatal breathing exercises for about 6 minutes and taught on one to one basis and were asked to practice these exercises daily twice a day and also continue during active phase of first stage of labor	home exercises, daily twice a day and continue during active phase of first stage of labor	assessed at 36 weeks of gestation and then followed up concurrently at the time of delivery (during the active phase of the first stage of labor	36 weeks of gestation	just once (video: 6 minutes and exercises explained)
Levett, 2016	natural state of relaxation (visualisation, breathing, massage, yoga), and facilitate labour progression (yoga, acupressure) and pain relief (breathing, acupressure, visualisation); education about the physiology of normal birth; partner support	pain relief through breathing; four breathing techniques were introduced: soft sleep breaths for relaxation between contractions; blissful belly breaths (BBs) which were used during contractions for pain relief; Cleansing Calming Breaths used following contractions during the transition period of labour; and the gentle birthing breath (GB) which was for use during the second stage of labour and encouraged descent of the baby avoiding active pushing and protection of the pelvic floor	2 days	Prior to 36 weeks	2 day course, once
Miquelutti, 2013	women participated in the physical and educational activities of the BPP conducted in addition to routine activities offered at the prenatal clinic, on the same days of the prenatal visits. During the meetings of approximately 50 minutes women performed non aerobic exercises of a protocol adapted for pregnancy and designed to attempt to reduce back pain, possibly to help venous return and to prevent UI and minimize anxiety. Participants received a guide with the exercises to be performed daily at home, consisting of: pelvic floor muscle training (PFMT) including rapid (30 times) and sustained maximal contractions (20 times holding for 10 seconds), stretching exercises to reduce back pain and exercises to improve venous return in the lower limbs.	Information about breathing exercises for delivery. Relaxation at home: Training of breathing techniques for contraction control during labor, Training of breathing techniques for contraction control during labor; progressive relaxation techniques; massage; mentalization	monthly from 18-24weeks till 30weeks; fortnightly till 36 weeks	18-24 weeks	Same days of prenatal visits on a monthly basis up to 30 weeks of pregnancy, fortnightly between 31 and 36 weeks of pregnancy and weekly from 37 weeks of preg-nancy onwards. Duration 50 minutes. Median of 5 meeting, range 2-10
Pan, 2019	MBCP by Nancy Bardack; transformative experience of pregnancy, childbirth, and postpartum-related adjustments in self-awareness training; listen to programme-related audio recordings at home six times a week for 30min each;		8 weeks	no information	Series of nine three-hour classes held once per week and one seven-hour day of silent-meditation practice. However: first- und second-week cours into one weel and time for each classe at three hour plus one seven-hour silent retreat. Additionally, listen to programme related audio recordings at home, six times a week for 30 min each
Prince, 2015	the experimental group were taught the selected antenatal exercises with the help of the video assisted program. The selected antenatal techniques that includeswere breathing exercises, relaxation exercises and pelvicfloor muscle exercise. The content that includes in the video assisted program are information about pregnancy and labor, benefits of selected antenatal exercise, typesof exercises, selected exercises during pregnancy and labor. Each primigravid women was given a record sheet to record the exercises performed at home with relevant instructions and a compact disk (CD) was provided for their practice. The video was done with lot of animation to make it easily understandable to the women.		34 weeks up to birth	no information	once, The session scheduled for 45 minutes and was asked to doa return demonstration by the primigravid women, and practice at home, <200 up to >=400 hours
Timm 1979	anatomical, emtional and physical changes in labour, relacation and chest-breathing patterns for use in labour, the delivery process, medications used in labour, emotinal and physical changes in postpartum, tour of hospital, combination of lectures, discussions, films and role-playing situations, use of self-learning programs available in waiting rooms		6 weeks	No information	10 series

Author, year	childbirth experience mean, SD (W-DEQ A/B)	childbirth self-efficacy mean, SD (CBSEI)	duration of labour	Memory of labour pain mean, SD (VAS)	Use of pain medication	Mode of birth	description of breathing technique	Duration of class	Frequency	
Abbasi 2021, 2018		Pre: IG software 141.8 (7.1) vs. IG booklet 143.3 (7.7) vs. CG 142.1 (7.5), p=0.563<br>Post: IG software 308.4 (11.3) vs. IG booklet 262.5 (39.5) vs CG 149.1 (23.0), p=<0.001		4cm: IG software 2.5 (0.8) vs. IG booklet 2.6 (0.8) vs. CG 2.6 (0.8), p=0.956<br>6cm: IG software 5.2 (0.7) vs. IG booklet 5.2 (0.5) vs. CG 5.1 (0.6), p=0.769<br>8cm: IG software 7.0 (0.8) vs. IG booklet 7.1 (0.8) vs. CG 7.1 (0.8), p=0.811<br>10cm: IG software 8.7 (0.9) vs. IG booklet 8.8 I0.7) vs. CG 8.7 (0.7), p=0.512						
Bergström, 2009	49 (26) vs. 50.1 (25), (mean diff -0.5, CI -3.2-4.1, p=1.0			4.9 (1.8) vs. 4.9 (1.8) (mean diff 0, CI -0.2-0.3, p=0.7)	epidural rate: 247 (52%) vs. 252 (52%) (RR 1.0, CI 0.9-1.1, p=0.9)	SG: 321 (66%) vs. 327 (66%);<br>Instrumental: 67 (14%) vs. 60 (12%),<br>I Sectio: 29 (6%) vs. 31 (6%),<br>II Sectio: 67 (14%) vs. 75 (15%)	**30 minutes** were spent on practical training in breathing; homework practicing breathing/relaxation	8 hours (four 2 hour sessions during pregnancy, 1 follow-up session within 10 weeks after delivery)	2hours weekly session	
Duncan, 2017	Pre: 67.1 (23.2) vs. 65.7 (11.9)<br>Post prior to birth: 58.0 (12.2) vs. 62.5 (13.0)<br>Post-birth: 57.1 (13.4) vs. 61.6 (20.8)	Pre: 165.1 (87.2) va. 197.3 (49.0)<br>Post Prior to birth: 243.3 (41.6) vs. 212.0 (35.4)			epidural rate: 12 (85.7) vs. 11 (84.6%)<br>(x2=0.01. df=1 p=0.94)<br>Opioid use: 4 (13) vs. 8 (13)		mindfulness breath, **practice at home** with audio and handouts	18 hours, 2.5 days, weekend workshop	A short, time-intensive 2.5-day weekend workshop	
Howarth, 2019		Post: 215.21 (38) vs. 190.81 (48) vs. TAU 180.61 (51)					exercises directed breathing, map pelvis, deep touch relaxation	40hours of contenct	50% had to be completed	
Karkada, 2017							**home exercises, daily twice a day** and continue during active phase of first stage of labor	assessed at 36 weeks of gestation and then followed up concurrently at the time of delivery (during the active phase of the first stage of labor	just once (video: 6 minutes and exercises explained)	
Levett, 2016			first stage: 6.12 (3.95) vs. 6.53 (3.9)<br>second stage: 1.00 (0.87) vs. 1.32 (0.98)<br>total lenght: 7.43 (4.13) vs. 8.20 (4.37)		epidural rate: 21 (23.9%) vs. 57 (68.7%)<br>(RR=0.35, CI=0.23-0.52, p=0.0001)	SG: 60 (68.2%) vs. 39 (47.0%)<br>Instrumental: 12 (13.6%) vs. 17 (20.5%)<br>II Sectio: 16 (18.2%) vs. 27 (32.5%)	pain relief through breathing; four breathing techniques were introduced: soft sleep breaths for relaxation between contractions; blissful belly breaths (BBs) which were used during contractions for pain relief; Cleansing Calming Breaths used following contractions<br>during the transition period of labour; and the gentle birthing breath (GB) which was for use during the second stage of labour and encouraged descent of the baby avoiding active pushing and protection of the pelvic floor	2 days	2 day course, once	
Miquelutti, 2013			first stage: 284.5 (175.0) vs. 254.2 (139.4)<br>second stage: 29.2 (23.3) vs. 19.7 (13.0)	lumbar: 5.1 (2.3) vs. 4.8 (2.5) (mean diff 0.34, CI -0.61-1.28)<br>pelvic: 5.5 (2.9) vs. 5.9 (2.8) (mean diff -0.38, -2.09-1.33)		SG: 44 (57.9%) vs. 38 (53.5%)	Information about breathing exercises for delivery. **Relaxation at home:** Training of breathing techniques for contraction control during labor, Training of breathing techniques for contraction control during labor; progressive relaxation techniques; massage; mentalization	monthly from 18-24weeks till 30weeks; fortnightly till 36 weeks	Same days of prenatal visits on a monthly basis<br>up to 30 weeks of pregnancy, fortnightly between 31 and<br>36 weeks of pregnancy and weekly from 37 weeks of preg-<br>nancy onwards. Duration 50 minutes. Median of 5 meeting, range 2-10	
Pan, 2019		Baseline: 229.33 (41.76) vs. 213.91 (44.67)<br>Stress: mean=2.82 (lower T1) and 2.79 lower p=0.01 (lower T2= p=0.01<br>CBSEI: mean score for the experimental group<br>was higher than that for the comparison group at baseline, this<br>difference was not significant (p = 0.07). However, the mean score<br>for the experimental group was 26.38 points (p = 0.001) higher<br>than the comparison group at T1 and 26.92 points higher<br>(p < 0.001) at T2.						8 weeks	Series of nine three-hour classes<br>held once per week and one seven-hour day of silent-meditation practice. However: first- und second-week cours into one weel and time for each classe at three hour plus one seven-hour silent retreat. Additionally, listen to programme related audio recordings at home, six times a week for 30 min each	After the intervention, the participants in the experimental group earned lower scores for stress and depression and higher scores for childbirth self-efficacy and mindfulness than their comparison group peers. These results support that stress affects variables that relate to depression and childbirth self-efficacy.
Prince, 2015				7.0 (1.0) vs. 8.8 (1.3) (t=19.65, p=<0.01)				34 weeks up to birth	**once**, The session scheduled for 45 minutes and was asked to doa return demonstration by the primigravid women, and **practice at home**, <200 up to >=400 hours	
Timm 1979			total lenght: 10.88 vs. 10.06 vs. 9.19 (no class)					6 weeks	10 series	

Author, year	1st min Apgar score >7	5th min Apgar score >7	birthweight > 2500grams	fetal blood sampling
Bergström, 2009				
Duncan, 2017				
Howarth, 2019				
Karkada, 2017			256 (95%) vs. 229 (92%) (OR=1.39, CI=0.68, p=0.36)	
Levett, 2016		3 (3.4%) vs. 4 (4.8%) (RR=0.99, CI=0.95-1.03, p=1.0)		
Miquelutti, 2013	70 (93.3%) vs. 63 (92.7%) (RR 1.01, CI 0.92-1.10)	75 (100%) vs. 67 (98.5) (RR 1.01, CI 0.99-1.04)	70 (92.1%) vs. 64 (94.1%) (RR 0.98, CI 0.90-1.07)	
Pan, 2019				
Prince, 2015				
Timm 1979				
