# Supplementary material for: The effectiveness of skilled breathing and relaxation techniques during antenatal education on maternal and neonatal outcomes: a systematic review
Source: BMC Pregnancy Childbirth. 2022 Nov 19;22:856. doi: 10.1186/s12884-022-05178-w (PMC9675115; doi:10.1186/s12884-022-05178-w)
Supplement: Supplementary file 2 — Additional file 2. [file 12884_2022_5178_MOESM2_ESM.docx]

**Search Strategy for a planned systematic Review**

| **Medline** | **Keywords** | **Matches** |
| --- | --- | --- |
|  | (childbirth education") AND "breathing techniques" | 2 |
|  | (("antenatal education") AND "breathing techniques") OR "relaxation" | 124834 |
|  | (((childbirth OR antenatal education)) AND "breathing exercises") OR "breathing techniques" | 389 |
|  | (breathing exercises[MeSH Terms]) AND "childbirth education" | 1 |
|  | ((breathing exercises[MeSH Terms]) AND "childbirth education") AND "childbirth outcomes" | 0 |
|  | ("breathing exercise") AND "childbirth education" | 0 |
|  | (("childbirth education") AND "breathing exercise") AND "childbirth outcomes" | 0 |
|  | ("childbirth education") AND breathing* | 10 |
|  | ((education program*) AND childbirth) AND breathing* | 4 |
|  | (((childbirth[MeSH Terms]) AND education program)) AND breathing | 5 |
|  | (childbirth[MeSH Terms]) AND education program | 196 |
| **CINHAL** | childbirth education AND breathing techniques OR breathing exercises | 2689 |
|  | childbirth education AND breathing techniques OR breathing exercises AND birth outcomes | 10 |
|  | childbirth education AND (breathing exercises or breathing techniques ) AND ( birth outcomes or pregnancy outcomes ) | 1 |
|  | antenatal education or prenatal education or antenatal classes or prenatal classes or birth preparation or childbirth classes ) AND ( breathing exercises or breathing techniques ) | 18 |
|  | **childbirth education* AND (breathing technique) OR (breathing exercise)** | 9344 |
| **ClinicalTrial.gov** | Pregnancy AND breathing | 98 |
| **Cochrane Library** | MeSH descriptor: [Prenatal Education] explode all trees and with qualifier(s): [methods - MT] | 25 |
|  | ("childbirth training"):ti,ab,kw AND (breathing technique):ti,ab,kw | 1 |
| **Embase** | ('pregnancy'/exp OR 'child bearing' OR 'childbearing' OR 'gestation' OR 'gravidity' OR 'intrauterine pregnancy' OR 'labor presentation' OR 'labour presentation' OR 'pregnancy' OR 'pregnancy maintenance' OR 'pregnancy trimesters') AND 'childbirth education'/exp AND 'breathing'/exp | 10 |
|  | ('pregnancy'/exp OR 'child bearing' OR 'childbearing' OR 'gestation' OR 'gravidity' OR 'intrauterine pregnancy' OR 'labor presentation' OR 'labour presentation' OR 'pregnancy' OR 'pregnancy maintenance' OR 'pregnancy trimesters') AND 'childbirth education'/exp | 460 |
| **MIDIRS** | (breath* and relax* and techniq* and birth) | 45 |
|  | ((breath* techniq* or breath*) and birth and outcome) | 170 |
|  | (( breathing exercises or breathing techniques ) AND ( birth OR labour OR labor ) AND ( heart rate OR pulse OR vital sign* )) | 0 |
|  | (relaxation technique* and (effect* or effectivenes*) and (birth or labour or labor or giving birth)) | 47 |
|  | (relaxation technique* and (effect* or effectivenes*) and (birth or labour or labor or giving birth) and (physical change* or bodyli change* or physical manifestation)) | 0 |
|  | ((labor or labour or birth* or giving birth or parturition) and (relax* or breath* or relaxation technique* or breathing technique*)) | 93969 |
|  | ((labor or labour or birth* or giving birth* or parturition) and (relax* or breath* or relaxation technique* or berathing technique*) and (exercise* or effect* or effectiveness* or physicalc change*)) | 79672 |
|  | ((labor or labour or birth* or giving birth* or parturition) and (relax* or breath* or relaxation technique* or berathing technique*) and (exercise* or effect* or effectiveness* or physicalc change*))¨  limit 2 to yr="2019 -Current" | 4550 |
|  | ((birth or parturition or labor or labour) and (relax* or breath*))  limit 6 to yr="2019 -Current" | 4890 |
|  | (berath* and relax* and technique* and birth*) | 0 |
